# Analysis of flavonoid metabolism of compounds in succulent fruits and leaves of three different colors of Rosaceae

**DOI:** 10.1038/s41598-024-55541-4

**Published:** 2024-02-28

**Authors:** Chen Yang, Nan Sun, Xin Qin, Yangbo Liu, Mengyi Sui, Yawen Zhang, Yanli Hu, Yunfei Mao, Xiang Shen

**Affiliations:** 1 College of Horticulture Science and Engineering, Shandong Agricultural University, Tai’an, 271000 China; 2 Hebei Agricultural University, College of Horticulture, Baoding, 071001 China

**Keywords:** Color pigmentation, Rosaceae, Metabolome, Flavonoids, Metabolomics, Plant sciences, Bioinformatics, Mass spectrometry

## Abstract

Red flesh apple (*Malus pumila var. medzwetzkyana Dieck*), purple leaf plum (*Prunus cerasifera Ehrhar f*), and purple leaf peach (*Prunus persica ‘Atropurpurea’*) are significant ornamental plants within the Rosaceae family. The coloration of their fruits and leaves is crucial in their appearance and nutritional quality. However, qualitative and quantitative studies on flavonoids in the succulent fruits and leaves of multicolored Rosaceae plants are lacking. To unveil the diversity and variety-specificity of flavonoids in these three varieties, we conducted a comparative analysis of flavonoid metabolic components using ultra-high-performance liquid phase mass spectrometry (UPLC-MS/MS). The results revealed the detection of 311 metabolites, including 47 flavonoids, 105 flavonols, 16 chalcones, 37 dihydroflavonoids, 8 dihydroflavonols, 30 anthocyanins, 14 flavonoid carbon glycosides, 23 flavanols, 8 isoflavones, 11 tannins, and 12 proanthocyanidins. Notably, although the purple plum and peach leaves exhibited distinct anthocyanin compounds, paeoniflorin and corythrin glycosides were common but displayed varying glycosylation levels. While the green purple leaf peach fruit (PEF) and red flesh apple leaf (AL) possessed the lowest anthocyanin content, they exhibited the highest total flavonoid content. Conversely, the red flesh apple fruit (AF) displayed the highest anthocyanin content and a diverse range of anthocyanin glycosylation modifications, indicating that anthocyanins predominantly influenced the fruit's color. Purple PLF, PLL, and PEL showcased varying concentrations of anthocyanins, suggesting that their colors result from the co-color interaction between specific types of anthocyanins and secondary metabolites, such as flavonols, flavonoids, and dihydroflavonoids. This study provides novel insights into the variations in tissue metabolites among Rosaceae plants with distinct fruit and leaf colors.

## Introduction

Rosaceae plants, like peaches, apples, and plums, are economically important fruits consumed worldwide^1^. Apart from their aesthetic appeal, they serve as vital sources of nutrition for humans and have been extensively studied for their nutritional and functional value^2^. Anthocyanin, a health-promoting pigment, imparts a dark color to Rosaceae fruits and leaves^3^. Anthocyanins find applications in food coloring, as well as in dyes, medicine, cosmetics, etc.^4^. Additionally, the diverse range of Rosaceae fruit types offers an excellent opportunity to study color formation and fruit breeding^5^.

Flavonoids, essential secondary metabolites, play a significant role in protecting plants against various stresses^6^. Flavonoids encompass yellow pigments derived from flavonoids, including chalcone, dihydroflavonoids, Flavones, and anthocyanins^7^. In plants, flavonoids primarily exist as glycosides bound to sugars or in free form^8^.

Flavonoids are important polyphenols synthesized in plants and are bioactive secondary metabolites, mainly include flavonoids, flavanones, isoflavones, flavonols, flavanones, anthocyanins, which are abundant in plants^9^. Flavonoids are widely distributed in plants, such as fruits, vegetables, tea and so on. According to its chemical structure, oxidation degree and unsaturation of connecting chain, it can be divided into flavones, flavanones, isoflavones, flavonols and anthocyanins^10^. Usually, different kinds of plants contain different types of dominant flavonoids. For example, flavones, such as luteolin and apigenin, are enriched in sweet pepper and celery respectively^11,12^; flavanones are mainly found in citrus fruits, including hesperidin and naringin^13^; isoflavones are mainly found in soybeans and legumes^14^; flavonols, including quercetin and rutin, are widely found in vegetables and fruits, with the highest content of red onion^15^; flavanols are mainly catechins, of which green tea is the most abundant^16^; anthocyanins determine the color of plants and are usually found in large quantities in colored stems, leaves, flowers and fruits^17^.

The high flavonoid content in Rosaceae fleshy fruits, especially as a natural source of anthocyanins^18^, contributes to their popularity. Recent studies have extensively investigated anthocyanins due to their potential health benefits, including antioxidant, anti-aging, anti-carcinogenic properties, and other biological activities^19^. Oxidative stress (OS) is considered an important factor causing aging and disease^20^. Anthocyanins belong to bioflavonoids, whose main physiological functions are free radical scavenging and antioxidant capacity. Studies have shown anthocyanins to be the most effective antioxidants and powerful free radical scavengers, as evident from their antioxidant activity being 50 and 20 times higher than Vitamin E (VE) and Vitamin C (VC), respectively^21^. Additionally, anthocyanin extracts at certain concentrations have demonstrated effective carcinogenesis prevention activity at varying stages^22^. In food applications, anthocyanins serve as nutritional fortifiers and natural, safe, and healthy preservatives^23^.While red flesh apple, purple leaf plum, and purple leaf peach, as ancient Rosaceae varieties, exhibit higher anthocyanin contents in fruits and leaves compared to common apples^24^, peaches^25^, and plums^26^, there is limited comparative research on flavonoids and antioxidant activity between succulent fruits and leaves among different Rosaceae plants. Flavonoid metabonomics is a valuable method for evaluating the comprehensive expression profile of flavonoids and studying subtle changes in hundreds of low-concentration metabolites.

This study utilized the succulent fruits and leaves of Rosaceae's red-fleshed apples, purple-leaf plums, and purple-leaf peaches as experimental materials to investigate the metabolic differences that may contribute to the distinct coloration of succulent fruits and leaves in the Rosaceae family. Ultra-high performance liquid chromatography–mass spectrometry (UPLC-MS) was used to detect flavonoid accumulation in these tissues. The findings of this study offer essential theoretical support for evaluating the content and mechanisms underlying the color formation of major flavonoid metabolites in Rosaceae plants, thereby guiding future breeding strategies.

## Materials and methods

### Sampling of plant materials and preparation of extracts

The fruit (AF) and leaf (AL) of the red flesh apple (*Malus pumila var. medzwetzkyana Dieck*), the fruit (PLF) and leaf (PLL) of the purple leaf plum (*Prunus cerasifera Ehrhar f*), and the fruit (PEF) and leaf (PEL) of the purple leaf peach (*Prunus persica ‘Atropurpurea’*) were collected from the Horticultural Experimental Station of Shandong Agricultural University. After collection, the tissue samples were rinsed with tap water and then sterilized water. Subsequently, they were frozen in liquid nitrogen and stored at – 80 °C in a refrigerator until further analysis. Each treatment comprised three biological repeats, with each repeat comprising five separate plant samples. No approvals were required for the study, which complied with all relevant regulations.

The preparation of the extract refers to the method described by Medda^27^ and is slightly improved. Fruits (pericarp and 1 mm pulp) and leaves are ground with liquid nitrogen. After grinding the plant tissue in an ice bath, the plant tissue was transferred to a 15 mL calibration test tube, and 0.1% HCl-ethanol solution was used to rinse the mortar and fix the volume. Ultrasonic extraction for 2 h, then 8000*g* room temperature centrifugation 10 min, the supernatant was taken to be determined. Each sample was extracted three times. The dry matter of 1 g fresh sample was determined after drying at 65 °C for 24 h.

### Determination of total flavonoids and anthocyanins

The contents of total flavonoids in fruits and leaves were determined by aluminum nitrate colorimetry and modified according to yang's method^28^. The content of total flavonoids was determined at 510 nm, and the content of flavonoids in the sample was determined with rutin (mg RE/g DW) as the control substance. The standard curve was established as follows: y = 5.02x + 0.0007, R^2^ = 0.9996. The results were expressed by the number of milligrams of rutin equivalent (rutin equivalent, RE) per gram of sample dry weight (dry weight, DW) (mg RE g^−1^DW).

The content of total anthocyanins was determined according to the method described by Lee^29^. The supernatant was mixed with pH 1 potassium chloride solution (0.025 M), and pH 4.5 sodium acetate solution (0.4 M) was added to the same supernatant to prepare blank. After incubation at room temperature for 30 min, the absorbance values at 520 and 700 nm were measured. The content was calculated by Lee's equation^29^. The results were expressed as anthocyanin 3-glucoside equivalent (C3G)/g DW mg.

### Sample preparation and extraction

The biological samples were prepared according to the method described by Chen et al.^30^. Freeze drying was carried out using a Scientz-100F vacuum freeze dryer, and then crushed at a speed of 30 Hz in a Retsch MM 400 mixer mill with zirconia beads.

According to the method of Yu et al.^31^. 100 mg freeze-dried powder was dissolved in 1.2 mL 70% methanol solution. The mixture was swirled for 30 s every 30 min and repeated for 6 times. The sample is then placed in a refrigerator at 4 °C overnight. After centrifugation at 12,000 rpm for 10 min, a SCAA-104 filter with an aperture of 0.22 μm (ANPEL, Shanghai, China) was used for filtration, and then UPLC-MS/MS analysis was carried out.

### UPLC conditions

The extract of the sample was analyzed by UPLC-ESI–MS/MS system of UPLC Nexera X2 (https://www.shimadzu.com.cn/) and the MS (Applied Biosystems 4500 Q TRAP, https://www.thermofisher.cn/cn/zh/home/brands/applied-biosystems.html). According to the He's method^32^. In the UPLC analysis, Agilent SB-C18 column (1.8 μm, 2.1 mm*100 mm) was used as the stationary phase, and the mobile phase included pure water containing 0.1% formic acid (solvent A) and acetonitrile containing 0.1% formic acid (solvent B). The sample was measured using a gradient program, use the gradient program to measure the sample, and the program is set up according to the description of Zhang et al.^33^, starting with a ratio of 95% A to 5% B. Within 9 min, adjust the scale to 5% An and 95% B through a linear gradient, and maintain the ratio for 1 min. Then, adjust the scale back to 95% An and 5% B within 1.1 min and maintain the ratio for 2.9 min. The flow rate is set to 0.35 mL/min, the temperature of the column oven is 40 °C, and the injection volume is 4 μL. The effluent is ionized and detected by connecting to an ESI-QTRAP-MS.

### ESI-Q TRAP-MS/MS

LIT and triple quadrupole (QQQ) scans were performed on the AB4500Q TRAP UPLC/MS/MS system. The system is equipped with ESI Turbo ion spray interface, which can work in positive and negative ion mode and is controlled by Analyst 1.6.3 software (AB Sciex). The operation parameters of the ESI source are described by Chong et al.^34^. In QQQ and LIT modes, we used 10 and 100 Mol/L polypropylene glycol aqueous solution for instrument tuning and mass calibration, respectively, We monitored a specific set of MRM transitions based on the metabolites eluted during each period^35^.

### Data processing and analysis

In this study, we used the metabolite database MWDB for qualitative determination of the substances in this study, and quantitatively analyzed the metabolites through the second-order spectral information and the MRM mode of triple quadrupole mass spectrometry.

#### PCA (principal component analysis) and HCA (hierarchical cluster analysis)

Use the statistical function prcomp in R (www.r-project.org) to carry out unsupervised PCA (principal component analysis). Before the unsupervised PCA, the data were measured by unit variance. The HCA (hierarchical cluster analysis) results of samples and metabolites are represented by the heat map of the tree map, while the Pearson correlation coefficient (PCC) between samples is represented by the cor function in R and presented in the form of heat map, R-packet premap was used in both HCA and PCC analysis^34^. For HCA, the normalized signal strength (unit variance scale) of metabolites is visualized as a chromatogram.

#### Differential metabolites selected

Analyze the metabolic group data according to the OPLS-DA model, draw the score chart of each group, further show the differences between each group, and analyze the metabolic group data according to the OPLS-DA model, draw the score chart of each group, and further show the difference between each group^31^. The screening of significantly regulated metabolites between groups was determined according to VIP ≥ 1 and absolute log_2_FC (folding change) ≥ 1. VIP values were extracted from OPLS-DA result, which also contain score plots and permutation plots, was generated using R package MetaboAnalystR. The data was log-transform (log_2_) and mean centering before OPLS-DA. In order to avoid overfitting, a permutation test (200 permutations) was performed^36^.

#### KEGG annotation and enrichment analysis

The identified metabolites were annotated by KEGG compound database (http://www.kegg.jp/kegg/compound/). The metabolites of these annotations are then mapped to the KEGG Path database (http://www.kegg.jp/kegg/pathway.html), Then, the pathway of significant up-regulation or down-regulation of metabolites was fed into the metabolite concentration analysis (MSEA), and its significance was determined by the p value of the hypergeometric test^37^.

### Plant guide statement

Our experimental and field studies on plants (whether cultivated or wild), including the collection of plant materials, comply with the International Union for Conservation of Nature Policy statement on Endangered species Research and the Convention on Trade in Endangered species of Wild Fauna and Flora.

## Results and analysis

### Phenotypic observation of fruits and leaves of three Rosaceae plants

**Figure 1:**
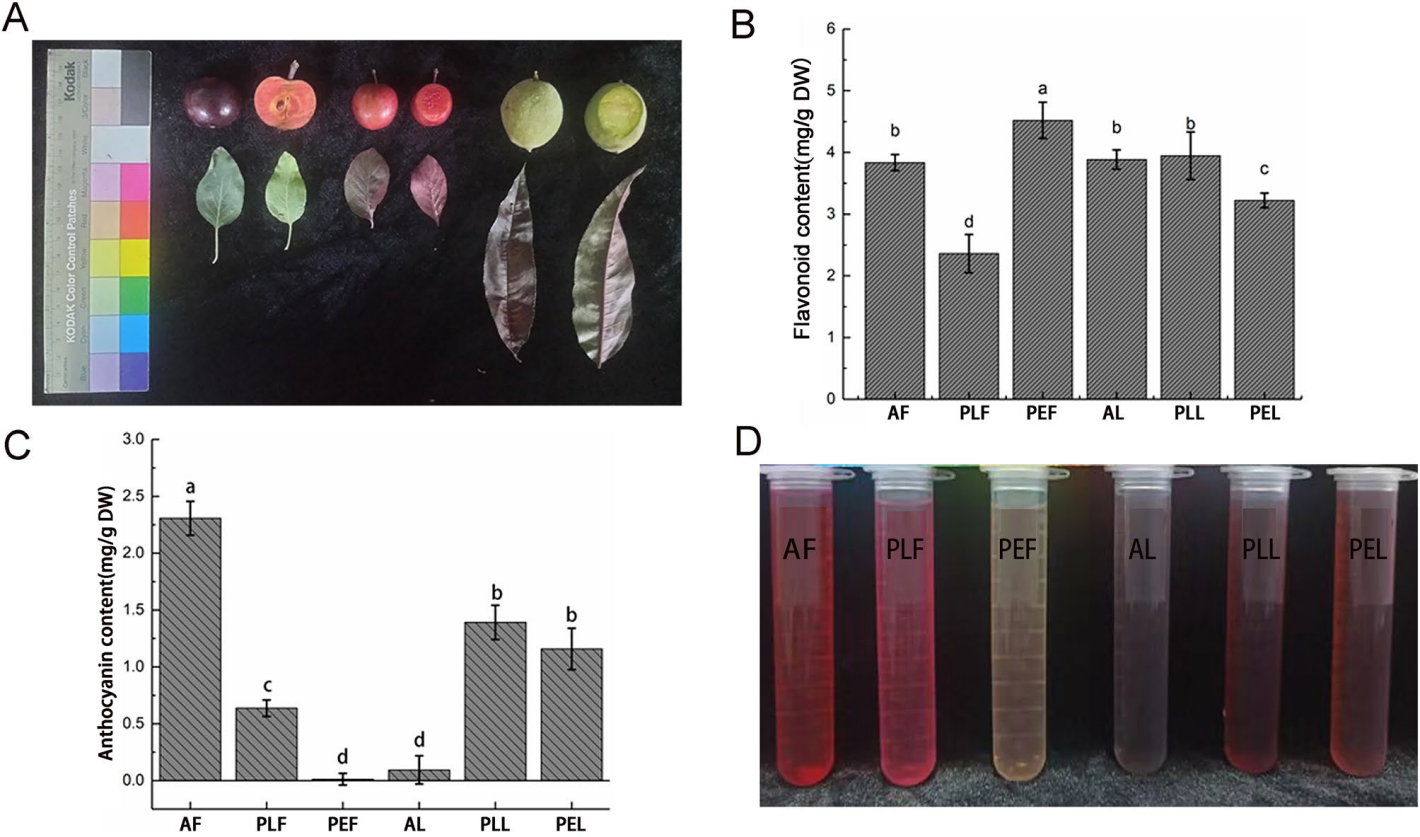
Content of flavonoids and anthocyanins and Phenotype in three Rosaceae plant materials. (**A**) Phenotype of AF, PEF, PLF in fruits and AL, PEL, PLL in leaves of three Rosaceae plants; (**B**) flavonoid contents; (**C**) anthocyanin contents; (**D**) tissue extract.

By examining mature fruits and complete leaves of red flesh apple, purple leaf plum, and purple leaf peach, it was observed that the fruit of red flesh apple and purple leaf plum had a purplish-red color, while the fruit of purple leaf peach was green. Interestingly, the leaves displayed different colors compared to the fruit. Specifically, the leaves of red flesh apple, purple leaf plum, and purple leaf peach were green, purple, and purple, respectively (Fig. 1A).

To further investigate this phenomenon, we determined the flavonoid and anthocyanin contents in six groups of plant materials. The results revealed that the flavonoid content in the fruit of purple leaf plum was significantly lower than those of the other five materials, whereas the fruit of purple leaf peach exhibited the highest flavonoid content. The flavonoid contents in the remaining four groups showed no significant differences (Fig. 1B).

Regarding anthocyanin content, red flesh apple fruit demonstrated significantly higher levels than the other groups. Following this, purple leaf plum leaf and purple leaf peach leaf displayed relatively high anthocyanin content, while the anthocyanin content in purple leaf peach fruit was the lowest (Fig. 1C).

Considering the phenotypic observations, a clear correlation emerged: the darker the plant material, the higher the anthocyanin content. Additionally, the light-colored fruits and leaves exhibited remarkably high flavonoid content, suggesting the possibility of metabolic differences between them (Fig. 1D).

### Metabolic analysis of fruits and leaves of three Rosaceae species

**Figure 2:**
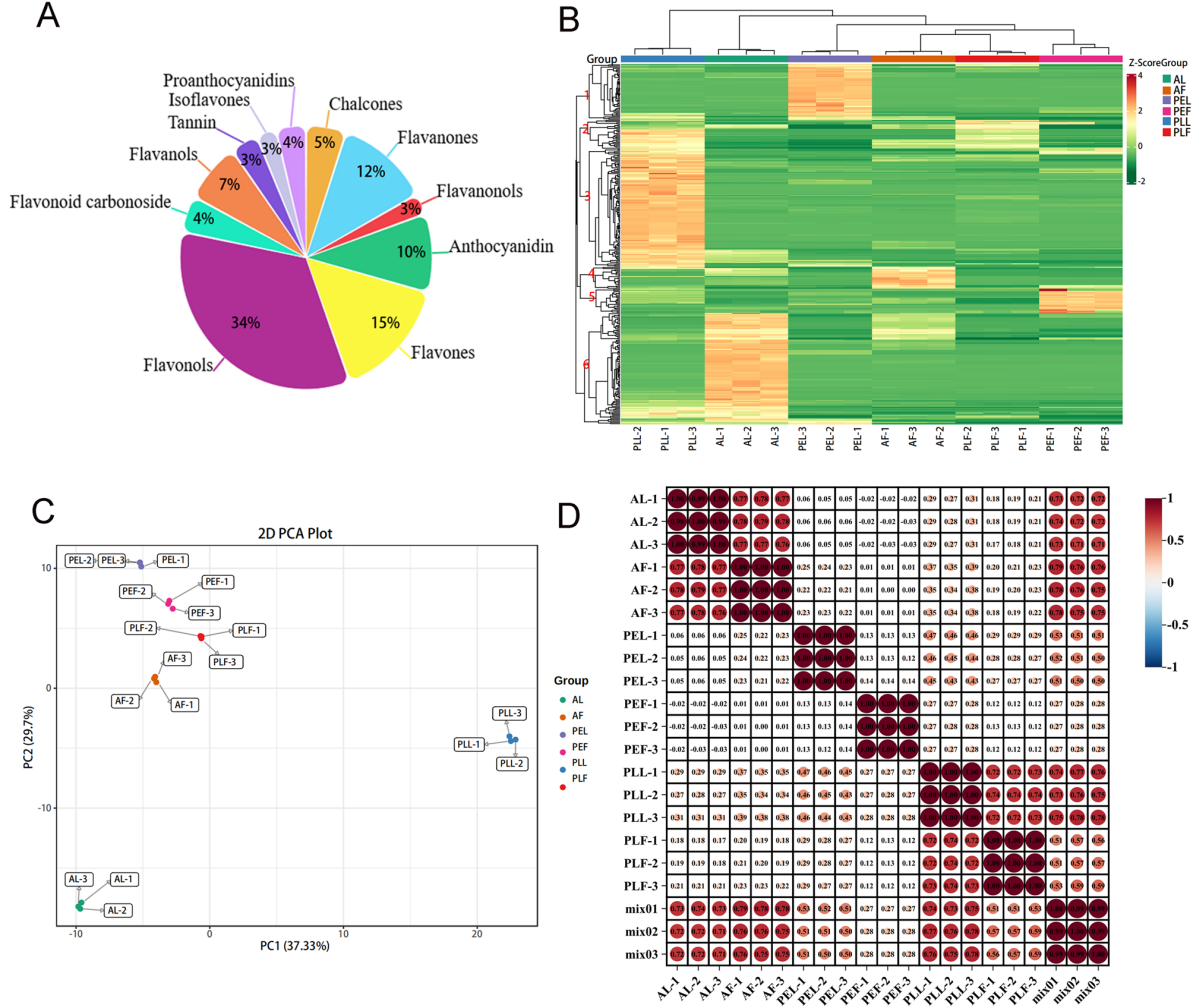
Metabolites analysis of fruits and leaves of three species of Rosaceae. (**A**) Classification pie chart of flavonoids; (**B**) differential metabolites clustering heat map. Horizontal for the sample name, vertical for the metabolite information, Group for the grouping, Class for the substance classification, different colors for the relative content standardized values (red for high content, green for low content), the clustering line on the left side of the figure is the metabolite clustering line, and the clustering line at the top of the figure is the sample clustering line; (**C**) principal component analysis (PCA); (**D**) correlation analysis diagram of differential metabolites. longitudinally and diagonally represent the sample names of different samples, different colors represent different Pearson correlation coefficients, the redder the color is, the stronger the positive correlation is, the whiter the color is, the worse the correlation is. The bluer the color is, the stronger the negative correlation is, and the size of the circle is proportional to the degree of correlation. At the same time, the correlation coefficient between the two samples is marked in the box.

The qualitative and quantitative analysis of metabolites and differential abundance metabolites (DAMs). components in six sample groups was performed using the widely targeted metabolic group of UPLC-MS/MS-based mass spectrometry. After quality evaluation, a total of 311 metabolites were detected, including 47 flavonoids, 105 flavonols, 16 chalcones, 37 dihydroflavonols, 8 dihydroflavonols, 30 anthocyanins, 14 flavonoid carbon glycosides, 23 flavanols, 8 isoflavones, 11 tannins, and 12 proanthocyanidins (Fig. 2A). HCA evaluation revealed differences in flavonoid metabolism among the six sample groups (Fig. 2B). Leaves exhibited higher enrichment levels (Species and relative content) of flavonoids than fruits, and the samples could be classified into six clusters based on the varying isoflavone enrichment levels. Cluster 1 showed the highest level of purple PEL, while cluster 2 had higher purple PLL accumulation. Cluster 3 showed the highest expression in purple PLF, followed by cluster 2. Cluster 4 had a significant accumulation of red AF, followed by cluster 6. Cluster 5 exhibited a notable accumulation of green PEF. Cluster 6 showed evident enrichment of green AL. This indicates different flavonoid accumulation patterns between the fruits and leaves of the three Rosaceae plants, with partial overlap of flavonoid metabolites within the same variety of plants.

PCA analysis (Fig. 2C) revealed distinct separation among the six sample groups, with closely gathered repeated samples. Three different regions could be distinguished, indicating variations in flavonoid metabolites in each region. The first group comprised purple PEL, green PEF, red PLF, and red AF, indicating similar flavonoid characteristics. The second group included purple PLL, and the third group included green AL. Therefore, although the results showed that the flavonoids in the fruits of the three Rosaceae plants were similar, they greatly differed in the leaves. The correlation heat map (Fig. 2D) also demonstrated good biological repetition among the six sample groups.

**Figure 3:**
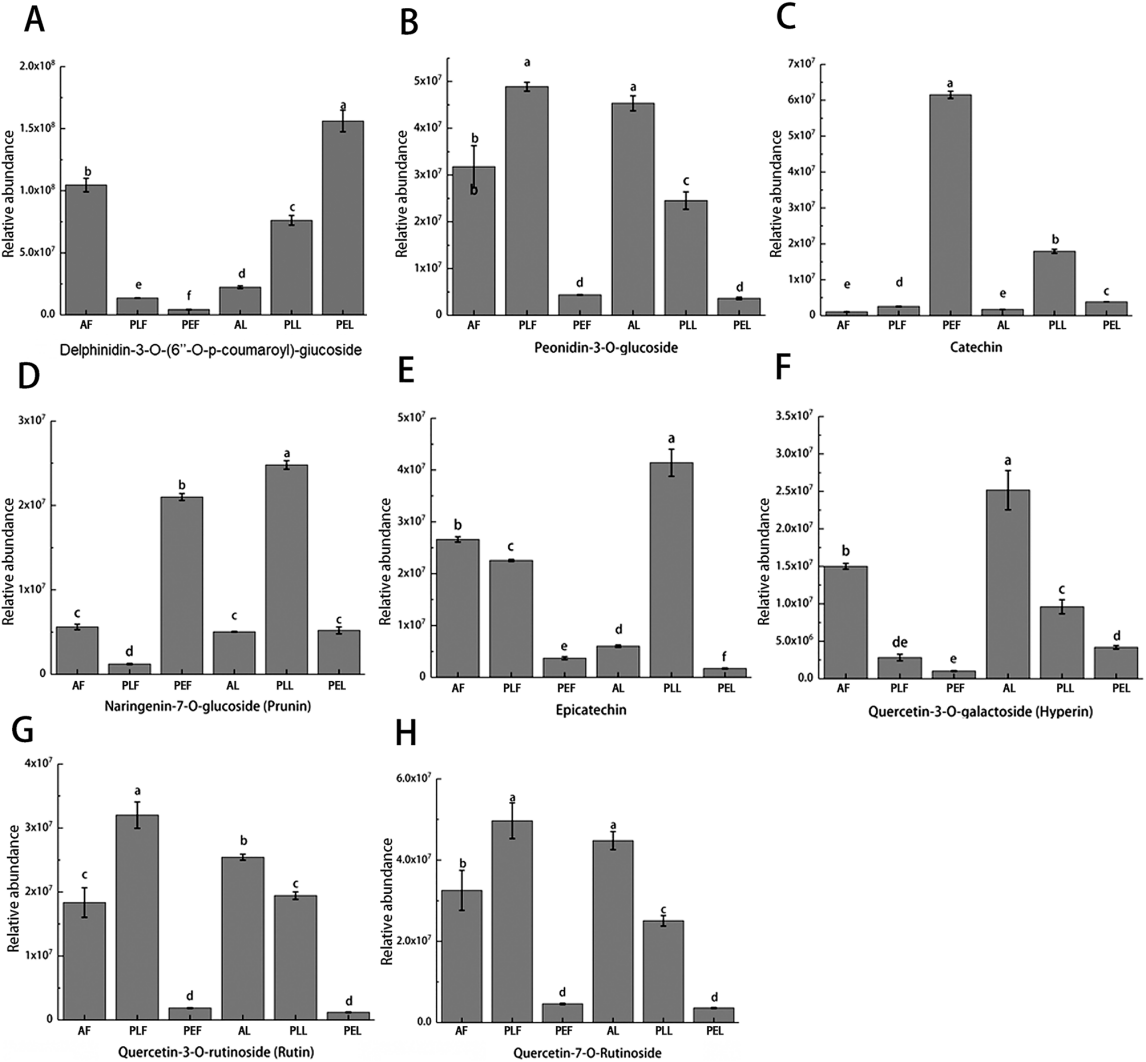
Relative abundance of anthocyanins and other flavonoids in the six groups of samples (**A**–**H**).

To further explore these differences, a preliminary analysis of the metabolites was conducted. The results showed modifications of quercetin, cypermethrin, kaempferol, luteolin, taraxanthin, hesperidin, apigenin, Tamariaxanthin, hesperidin, halcyrhamneol, and isorhamnetin through various glycosidic bonds in fruits and leaves of different colors. Glycosylation occurred at positions 8, 6, 3, 5, and 7, including neo-hesperidin, glucoside, isorhamnose, galactose, rutin, and glucuronide glycosylation. Eight flavonoids, namely Delphinidin-3-O-(6″-O-p-coumaroyl) glucoside, Peonidin-3-O-glucoside, Catechin, Naringenin-7-O-glucoside (prunin), Epicatechin, Quercetin-3-O-galactoside (Hyperin), Quercetin-3-O-rutinoside (Rutin) and Quercetin-7-O-Rutinoside, were co-enriched and exhibited high relative abundance in the six sample groups (Fig. 3A–H).

### Screening of DAMs in the fruits and leaves of three Rosaceae species

**Figure 4:**
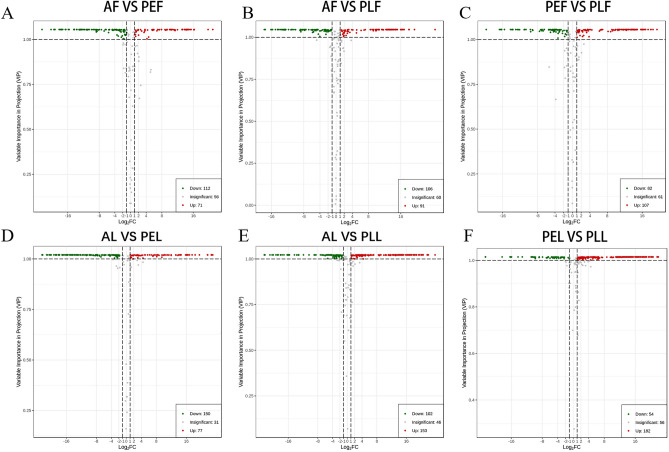
Volcanic diagram of DAMs in fruits and leaves of three Rosaceae plants (**A**–**F**). Each point in the volcanic map represents a metabolite, in which the green dot represents the down-regulated differential metabolite, the red dot represents the up-regulated differential metabolite, and the gray represents the metabolite detected but not significantly different; the Abscissa represents the logarithm of the multiple of the relative content difference between the two groups of samples (log_2_FC), the ordinate represents the VIP value, and the screening criterion is VIP ≥ 1 and absolute log_2_FC (fold change) ≥ 1.

DAMs were screened using the criteria of VIP ≥ 1 and log2FC (folding change) ≥ 1. A total of 183 DAMs (71 accumulated and 112 reduced) were identified between red AF and green PEF (Fig. 4A). Among them, 15 flavonoids and 15 dihydroflavonoids were accumulated, while 51 flavonols showed significant down-regulation. Between red AF and red PLF (Fig. 4B), there were 197 DAMs (91 accumulated and 106 reduced), with 29 flavonols accumulated and 38 reduced. To comparw green PEF and red PLF, 189 DAMs (107 accumulated and 82 reduced) were found (Fig. 4C). Among them, 43 flavonols were significantly accumulated, while 21 dihydroflavonols and 18 flavonols were significantly reduced. Additionally, between green AL and purple PEL (Fig. 4D), 227 DAMs (77 accumulated and 150 reduced) were identified. Among them, 20 dihydroflavonoids and 16 flavonols were significantly accumulated, while 67 flavonols showed significant down-regulation. Between green AL and purple PLL, there were 255 significantly accumulated and reduced DAMs (Fig. 4E). Specifically, 41 flavonols, 23 flavonoids, and 19 anthocyanins were significantly accumulated, while 45 flavonols were significantly reduced. To compare purple PEL and purple PLL (Fig. 4F), 236 DAMs (182 accumulated and 54 reduced) were observed. Among them, 75 flavonols and 19 anthocyanins were significantly accumulated, while 19 dihydroflavonoids showed significant down-regulation. In general, most differential metabolites in the six sample groups were flavonols, flavonoids, dihydroflavonoids, and anthocyanins. These compounds displayed a certain complementary trend within each sample. Compared to green PEF and AL, dark fruits and leaves exhibited lower levels of most flavonoids (flavonols, flavonoids, and dihydroflavonoids), while the contents of certain anthocyanins were higher.

### Changes of relative anthocyanin abundance in fruits and leaves of three different Rosaceae plants

The formation of plant color is influenced by the content and type of anthocyanins. To deepen our understanding, We made an in-depth analysis of the anthocyanin compounds with significant changes in six groups of samples (Table 1). A comparison between red AF and green PEF revealed that the fold change (FC) of 5 different anthocyanins ranged from − 22.57 to 20.97 (3 accumulated and 2 reduced). The three most accumulated anthocyanins were Cyanidin-3-O-Rutinoside (Keracyanin) (20.97-fold), Cyanidin-3-O-(2″-O-glucosyl) glucoside (14.64-fold), and Peonidin-3-O-(6″-O-p-coumaroyl) glucoside (14.56-fold), The most reduced anthocyanin was Peonidin-3-O-arabinoside (− 22.57-fold). Between red AF and purple PLF, 4 different anthocyanins were identified. The three most accumulated glucosides had fold changes of 24.48, 18.01, and 16.10, respectively. The most significant downregulation was observed in Cyanidin-3,5-O-diglucoside (Cyanin) (17.77-fold). Three different anthocyanins were detected between purple PLF and green PEF. Among the top 10 accumulated DAMs, two anthocyanins, Cyanidin-3-O-(2″-O-xylosyl) rutinoside (18.01-fold) and Delphinidin-3-O-(2‴-O-p-coumaroyl) rutinoside (16.04-fold), showed high enrichment. Petunidin-3-O-arabinoside (16.73-fold) was significantly reduced.

**Table 1:** Different cumulative metabolites among different comparison groups.

Group	Compounds	Class	VIP	p_value	FDR	Fold_Change	Log2FC	Type
AF_VS_PEF	Peonidin-3-O-arabinoside	Anthocyanidins	1.05	0	0.01	0	− 22.57	Down
	Cyanidin-3,5-O-diglucoside (Cyanin)	Anthocyanidins	1.05	0	0.01	0	− 17.77	Down
	Cyanidin-3-O-Rutinoside (Keracyanin)	Anthocyanidins	1.05	0	0	2,053,444.44	20.97	Up
	Cyanidin-3-O-(2″-O-glucosyl) glucoside	Anthocyanidins	1.05	0	0.01	25,548.52	14.64	Up
	Peonidin-3-O-(6″-O-p-coumaroyl) glucoside	Anthocyanidins	1.05	0	0.01	24,092.96	14.56	Up
AF_VS_PLF	Cyanidin-3,5-O-diglucoside (Cyanin)	Anthocyanidins	1.05	0	0.01	0	− 17.77	Down
	Cyanidin-3-O-Rutinoside (Keracyanin)	Anthocyanidins	1.05	0	0	23,410,740.7	24.48	Up
	Cyanidin-3-O-(2″-O-xylosyl) rutinoside	Anthocyanidins	1.05	0	0	264,614.81	18.01	Up
	Cyanidin-3-O-(2″-O-glucosyl) glucoside	Anthocyanidins	1.05	0	0.01	70,240	16.1	Up
PLF_VS_PEF	Petunidin-3-O-arabinoside	Anthocyanidins	1.06	0	0	0	− 16.73	Down
	Cyanidin-3-O-(2″-O-xylosyl) rutinoside	Anthocyanidins	1.06	0	0	264,614.81	18.01	Up
	Delphinidin-3-O-(2‴-O-p-coumaroyl) rutinoside	Anthocyanidins	1.06	0	0.01	67,544.44	16.04	Up
AL_VS_PEL	Peonidin-3-O-(6″-O-p-coumaroyl) glucoside	Anthocyanidins	1.02	0	0	7,141,518.52	22.77	Up
	Cyanidin-3-O-Rutinoside (Keracyanin)	Anthocyanidins	1.02	0	0	7,041,888.89	22.75	Up
	Cyanidin-3-O-(6″-O-caffeoyl) glucoside	Anthocyanidins	1.02	0	0	3,278,629.63	21.64	Up
AL_VS_PLL	Cyanidin-3-O-Rutinoside (Keracyanin)	Anthocyanidins	1.02	0	0	14,570,000	23.8	Up
	Cyanidin-3-O-(2″-O-xylosyl) rutinoside	Anthocyanidins	1.02	0	0	813,311.11	19.63	Up
PLL_VS_PEL	Cyanidin-3-O-(6″-O-feruloyl) sophoroside-5-O-Glucoside	Anthocyanidins	1.01	0.01	0.01	0	− 15.93	Down
	Cyanidin-3,5-O-diglucoside (Cyanin)	Anthocyanidins	1.01	0.01	0.01	1,594,666.67	20.6	Up
	Cyanidin-3-O-(2″-O-xylosyl) rutinoside	Anthocyanidins	1.01	0	0	813,311.11	19.63	Up
PLF_VS_PLL	Cyanidin-3,5-O-diglucoside (Cyanin)	Anthocyanidins	1.04	0.01	0.01	1,594,666.67	20.6	Up
PEF_VS_PEL	Petunidin-3-O-arabinoside	Anthocyanidins	1.03	0	0	0	− 16.73	Down
	Cyanidin-3-O-(6″-O-feruloyl) sophoroside-5-O-Glucoside	Anthocyanidins	1.03	0.01	0.01	62,544.44	15.93	Up
	Peonidin-3-O-arabinoside	Anthocyanidins	1.03	0	0	34,590.74	15.08	Up

Between green AL and purple PEL, the top three accumulated anthocyanins were Peonidin-3-O-(6″-O-p-coumaroyl) glucoside (22.77-fold), Cyanidin-3-O-Rutinoside (Keracyanin) (22.75-fold), and Cyanidin-3-O-(6″-O-caffeoyl) glucoside (21.64-fold), while no significantly reduced anthocyanins were found.

For green AL and purple PLL, the two most accumulated anthocyanins were Cyanidin-3-O-Rutinoside (Keracyanin) (23.8-fold) and Cyanidin-3-O-(2″-O-xylosyl) rutinoside (19.63-fold). Between purple PEL and purple PLL, 3 differential anthocyanins were detected. The two most enriched anthocyanins were Cyanidin-3,5-O-diglucoside (Cyanin) (20.60-fold) and Cyanidin-3-O-(2″-O-xylosyl) rutinoside (19.63-fold), while “Cyanidin-3-O-(6″-O-feruloyl) sophoroside-5-O-Glucoside” was significantly decreased (− 15.93-fold). Among the three comparative combinations, AFvs.AL, PLFvs.PLL, and PEFvs.PEL only the PLFvs.PLL, and PEFvs.PEL comparison showed significant upregulation in three anthocyanins, namely Cyanidin-3,5-O-diglucoside (Cyanin) (20.6-fold), Cyanidin-3-O-(6″-O-feruloyl) sophoroside-5-O-Glucoside (15.93-fold) and Peonidin-3-O-arabinoside (15.08-fold). Morning Petunidin-3-O-arabinoside (− 16.73-fold) was significantly reduced. These results demonstrate significant differences in anthocyanin content between fruits and leaves of different Rosaceae varieties. Moreover, even slight variations in anthocyanins within the same variety can result in noticeable differences in color.

**Figure 5:**
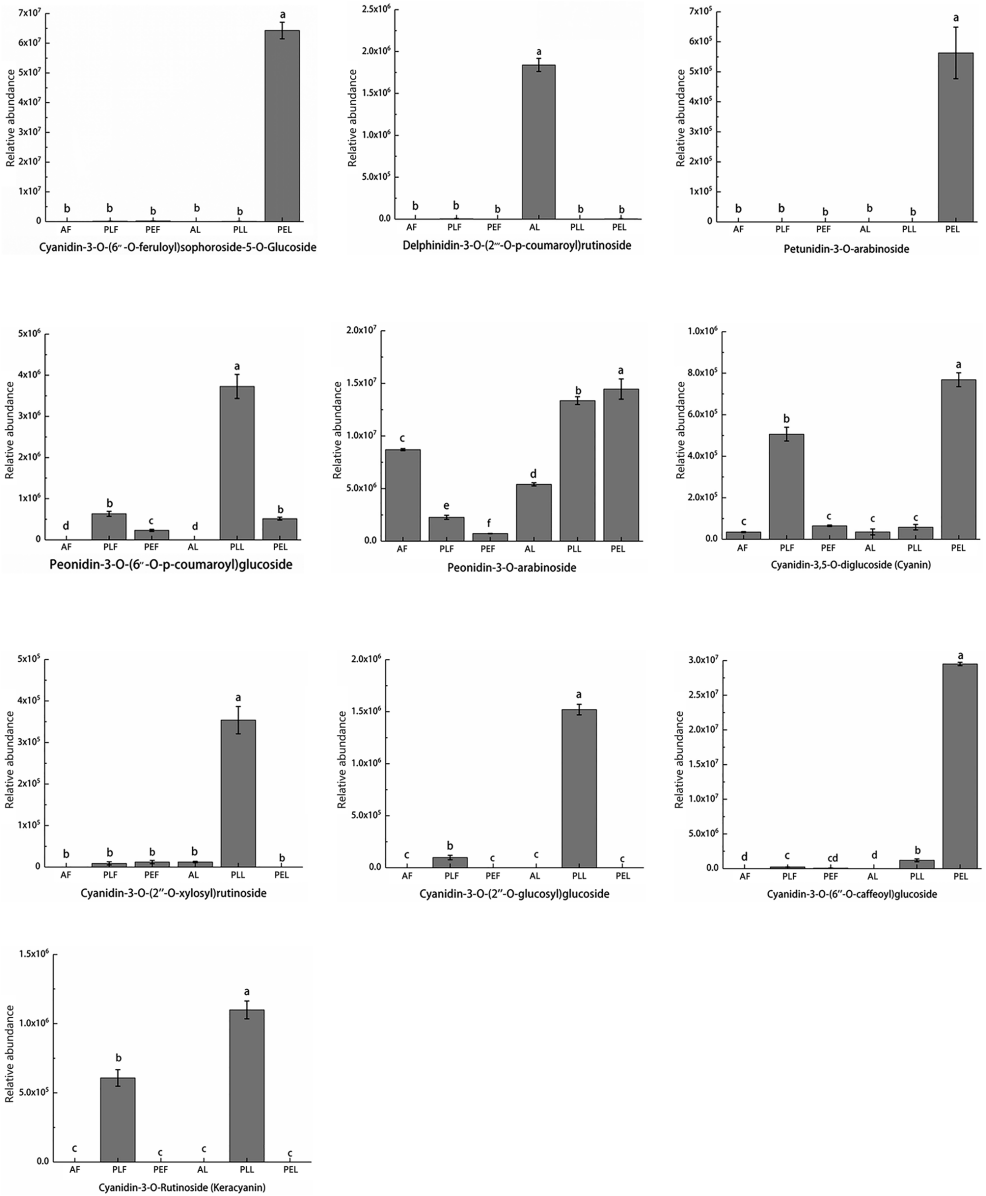
Relative abundance of ten anthocyanins detected in all samples.

Figure 5 illustrates the relative content of representative anthocyanins in each sample. The main representative anthocyanins include Cyanidin-3,5-O-diglucoside (Cyanin), Cyanidin-3-O-(2″-O-glucosyl) glucoside, Cyanidin-3-O-(2″-O-xylosyl) rutinoside, Cyanidin-3-O-(6″-O-caffeoyl) glucoside, Cyanidin-3-O-Rutinoside (Keracyanin), Delphinidin-3-O-(2‴-O-p-coumaroyl) rutinoside, Peonidin-3-O-(6″-O-p-coumaroyl) glucoside, Peonidin-3-O-arabinoside and Petunidin-3-O-arabinoside. These anthocyanins exhibit varying levels in the six groups of samples. The compound “Cyanidin-3-O-(6″-O-feruloyl) sophoroside-5-O-Glucoside”showed significant enrichment only in PEL, and the highest abundance of Peonidin-3-O-arabinoside, Petunidin-3-O-arabinoside, Cyanidin-3-O-(6″-O-caffeoyl) glucoside, and Cyanidin-3,5-O-diglucoside (Cyanin) was also observed in PEL.

Furthermore, Delphinidin-3-O-(2‴-O-p-coumaroyl) rutinoside exhibited significant enrichment in AL. PLL had the highest contents of Peonidin-3-O-(6″-O-p-coumaroyl) glucoside, Peonidin-3-O-arabinoside, Cyanidin-3-O-(2″-O-xylosyl) rutinoside, Cyanidin-3-O-(2″-O-glucosyl) glucoside and Cyanidin-3-O-Rutinoside (Keracyanin). Peonidin-3-O-arabinoside and Cyanidin-3,5-O-diglucoside (Cyanin) had higher levels in AF and PLF, respectively. PLL, PEL, and AF exhibited the highest abundance of Peonidin-3-O-arabinoside.

### Key differential metabolites in leaves and fruits of different colors

**Figure 6:**
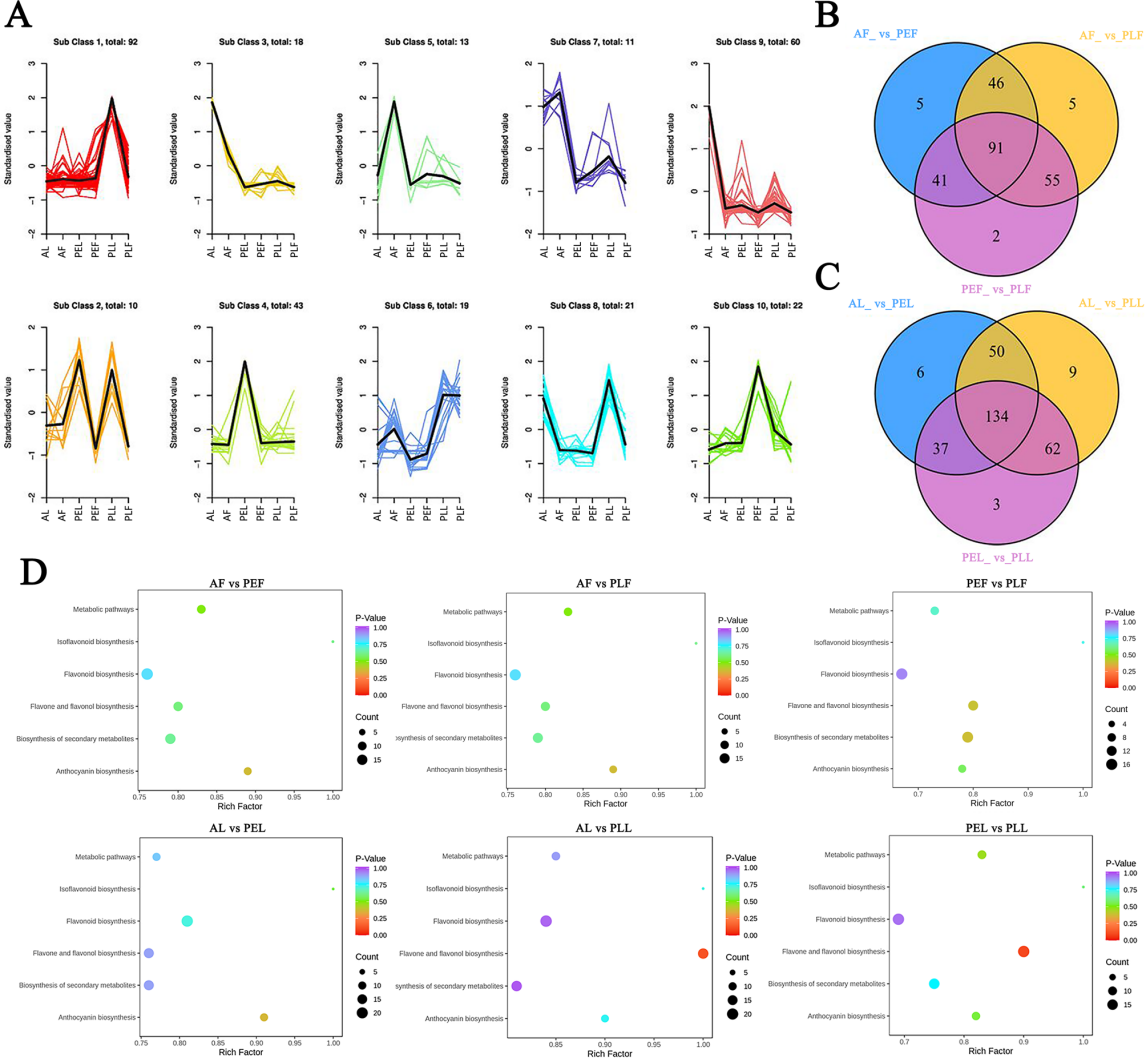
k-means analysis (**A**), Venn diagram (**B**,**C**) and KEGG analysis (**D**) of DAMs in six groups of samples. (**A**) Abscissa represents the name of the sample, ordinate represents the standardized relative content of metabolites, Sub Class represents the class number of metabolites with the same trend, and total represents the number of metabolites in this category. (**B**) In the figure, each circle represents a comparison group, the number of circle and circle overlap represents the number of differential metabolites shared between the comparison groups, and the number without overlap represents the number of unique differential metabolites of the comparison group. (**D**) Abscissa represents the corresponding Rich Factor of each path, ordinate is the name of the path, and the color of the point reflects the size of p value, and the redder it is, the more significant the enrichment. The size of the point represents the number of differential metabolites enriched.

In order to determine the relationship between different metabolites and six groups of plant materials, we conducted the k-means analysis and found that they can be divided into 10 clusters (Fig. 6A). Cluster 1 contained 92 metabolites, cluster 2 had 10 metabolites, cluster 3 had 18 metabolites, cluster 4 had 43 metabolites, cluster 5 had 13 metabolites, cluster 6 had 19 metabolites, cluster 7 had 11 metabolites, cluster 8 had 21 metabolites, cluster 9 had 60 metabolites, and cluster 10 had 22 metabolites The metabolites in PLL had the highest peak in clusters 1, 2, 6, 7, and 8, while PEL had a higher representation in clusters 2 and 4. AF had the highest representation in clusters 5, 6, and 7, and AL was highly enriched in clusters 3, 7, 8, and 9.

A total of 245 differential substances were found in the three comparison groups of AFvs.PEF, AFvs.PLF, and PEFvs.PLF, with 91 overlapping substances (Fig. 6B). These differential substances consisted mainly of 81 flavonols, 32 flavonoids, 30 dihydroflavonoids, and 23 anthocyanins. In the comparison groups of AL vs PEL, AL vs PLL, and PEL vs PLL (Fig. 6C), there were 134 differentials. A total of 221 substances were identified. Among these, 105 were flavonols, 45 were flavonoids, 36 were dihydroflavonoids, and 29 were anthocyanins.

Analysis of the KEGG pathway annotation and enrichment revealed that the differential metabolites were involved in various pathways, including flavonoid biosynthesis, metabolic pathway, secondary metabolite biosynthesis, isoflavone biosynthesis, flavonoid and flavonol biosynthesis, and anthocyanin biosynthesis pathway (Fig. 6D).

## Discussion

Red-flesh apples, purple leaf plums, and purple leaf peaches are three types of ornamental fruit trees widely utilized in various green spaces due to their vibrant fruit and leaf colors^38^. Additionally, their roots and leaves possess medicinal properties with specific pharmacological activities. Combining both ornamental and medicinal value into one gives these trees broad application potential^39,40^. Colored plant tissues are rich in flavonoids and anthocyanins, which offer various physiological benefits to plants^41^. Studies have shown that flavonoids can enhance antioxidant capabilities and free radical scavenging in animals, thereby playing a crucial role in improving human health^42,43^.

Flavonoids are a kind of natural compounds which exist widely in plants and have a variety of biological activities and pharmacological effects^44,45^. A large number of studies have confirmed the importance of flavonoids in plants. For example, flavonoids can help plants resist adverse environmental conditions. Some studies have shown that flavonoids can enhance the antioxidant capacity of plants and reduce the damage caused by harmful substances such as ultraviolet rays and free radicals^46^. In addition, it has been reported that flavonoids can also improve the disease resistance of plants^47^. Other studies have shown that flavonoids have significant effects on plant growth and development, and can promote plant growth and development of plants, especially in the development of underground organs^48^. And because some flavonoids have a variety of biological activities, such as antioxidant, anti-inflammatory and cholesterol-lowering effects^49,50^, it has also been widely reported that they are suitable for use as natural medicines or health products.

Anthocyanin is a water-soluble natural pigment and a subtype of bioflavonoids that determines the color of most flowers, fruits, and seeds^51–53^. Different combinations of flavonoids not only generate distinct colors but also produce varying levels of antioxidant capacity^54,55^. Hence, determining flavonoid metabolic groups is essential for accurately understanding the content and types of flavonoids in each fruit and leaf. Flavonoid metabonomics studies demonstrated that the primary metabolites in all samples were flavonols, flavonoids, dihydrochalcone, and anthocyanins, with each group of samples emphasizing different flavonoid expressions and accumulation patterns. The fact that flavonols are highly concentrated in all samples confirms their shared chromogenic effect with anthocyanins.

Prior studies have indicated that the color change from purple to blue is related to the co-pigment system formed by anthocyanins and other flavonoids^56,57^. The color of anthocyanins in red plant tissues is primarily influenced by the degree of molecular modification, including glycosylation, methylation, and acylation^58,59^, which aligns with our findings. Moreover, the type and quantity of anthocyanins often vary across different colors and plant tissues. For instance, delphinidin (Dp) and anthocyanins (Cy) determine the blue and red colors of *senecio cruentus*, while pink flowers contain derivatives of anthocyanins (Cy) and geranitin (Pg). Purple flowers contain derivatives of Delphinidin and Cyanidin^60^. Additionally, pelargonin glycoside serves as the primary pigment source in strawberries^61^, and the synthesis and accumulation of piperidin and its derivatives play a pivotal role in the blue-purple pigmentation of most petals. By comparing the differential metabolites in fruits and leaves, it was observed that dark fruits and leaves exhibited higher levels of anthocyanins, while light-colored fruits and leaves contained higher levels of total flavonoids.

The abundance of Peonidin-3-O-glucoside was highest in all plant samples, with significant differences among groups. This suggests that Peonidin-3-O-glucoside may be a key factor influencing the color variation among the six groups of samples. Cyanidin-3-O-(6″-O-feruloyl)sophoroside-5-O-Glucoside, Petunidin-3-O-arabinoside, Cyanidin-3,5-O-diglucoside (Cyanin), and Cyanidin-3-O-(6″-O-caffeoyl)glucoside were significantly enriched only in PEL but were very low in PEF. Therefore, these substances are the primary reasons for the purple color of PEL. Surprisingly, AF exhibited a high concentration of anthocyanins, suggesting that the high glycosylation level of anthocyanins primarily influenced the color of red meat apples. Green coloration is associated with high flavonoid and low anthocyanin contents, as evident from the total flavonoids and anthocyanin levels in AL and PEF. Additionally, dark plant tissues are influenced not only by molecular modifications of anthocyanins but also by co-pigmentation. The composition and content of upstream flavonoid metabolites, such as catechins, kaempferol, quercetin, naringin, and isorhamnetin and their glycosides, are also related to color formation in dark tissues^62,63^. We found that purple PLF, PLL and PEL have higher levels of anthocyanins and more abundant anthocyanin glycosylated derivatives, and these three plants also show high levels of flavonoids, indicating that purple is mainly formed by the interaction of different types of anthocyanins with other secondary metabolites.

## Conclusion

The metabolites involved in fruit and leaf color variation are associated with various metabolic pathways, including tricarboxylic acid cycle intermediates. Anthocyanins undergo various glycosylation modifications mediated by flavonoid glycosyltransferases. This study revealed distinct color mechanisms between succulent fruits and leaves of three Rosaceae plants. Specifically, red plant tissues are primarily influenced by high levels of molecular modifications in anthocyanins, while purple plant tissues are mainly influenced by the hyperchromic effect of a few anthocyanins and various secondary metabolites.

### Supplementary Information

Supplementary Information.

## Data Availability

The original contributions presented in the study are publicly available.
